# Heatwave resilience of juvenile white sturgeon is associated with epigenetic and transcriptional alterations

**DOI:** 10.1038/s41598-023-42652-7

**Published:** 2023-09-18

**Authors:** Madison L. Earhart, Tessa S. Blanchard, Nicholas Strowbridge, Ravinder Sheena, Clark McMaster, Benjamin Staples, Colin J. Brauner, Daniel W. Baker, Patricia M. Schulte

**Affiliations:** 1 Department of Zoology, University of British Columbia, Vancouver, Canada; 2 School of Biodiversity, One Health, and Veterinary Medicine, College of Medical, Veterinary and Life Sciences, University of Glasgow, Glasgow, UK; 3 Department of Fisheries and Aquaculture, Vancouver Island University, Nanaimo, Canada

**Keywords:** Climate-change ecology, Physiology, DNA methylation, Conservation biology, Animal physiology, Transcriptomics, Molecular ecology

## Abstract

Heatwaves are increasing in frequency and severity, posing a significant threat to organisms globally. In aquatic environments heatwaves are often associated with low environmental oxygen, which is a deadly combination for fish. However, surprisingly little is known about the capacity of fishes to withstand these interacting stressors. This issue is particularly critical for species of extreme conservation concern such as sturgeon. We assessed the tolerance of juvenile white sturgeon from an endangered population to heatwave exposure and investigated how this exposure affects tolerance to additional acute stressors. We measured whole-animal thermal and hypoxic performance and underlying epigenetic and transcriptional mechanisms. Sturgeon exposed to a simulated heatwave had increased thermal tolerance and exhibited complete compensation for the effects of acute hypoxia. These changes were associated with an increase in mRNA levels involved in thermal and hypoxic stress (*hsp90a, hsp90b, hsp70* and *hif1a*) following these stressors. Global DNA methylation was sensitive to heatwave exposure and rapidly responded to acute thermal and hypoxia stress over the course of an hour. These data demonstrate that juvenile white sturgeon exhibit substantial resilience to heatwaves, associated with improved cross-tolerance to additional acute stressors and involving rapid responses in both epigenetic and transcriptional mechanisms.

## Introduction

Heatwaves, prolonged but discrete periods of unusually high temperatures, pose a major threat to organisms, ecosystem health, and global biodiversity^1–3^. In aquatic environments, increases in temperature often co-occur with decreased oxygen (hypoxia) as a result of multiple interacting mechanisms^4–6^, and these events are particularly challenging for aquatic ectotherms. For example, warming increases the rate of biological reactions, increasing aerobic metabolic demand, which necessitates an increase in oxygen supply to maintain energetic balance at a time when aquatic oxygen levels are in decline^4,6–8^. Exposures to the deadly combination of high temperature and hypoxia associated with heatwaves are likely to become more frequent, longer lasting, and more extreme across a wider range of aquatic habitats as climate change worsens^9–11^.

During an extended heatwave, organisms may use phenotypic plasticity to help offset the effects of increased temperatures. In many fish species, acclimation to warm conditions results in a number of beneficial changes including increased thermal tolerance^8^; but whether this plasticity also induces increased tolerance to hypoxia is unclear^12^. The ability to produce this cross-tolerance may be critically important because acute exposure to high temperature reduces hypoxia tolerance, and acute exposure to low oxygen reduces thermal tolerance^8^. Whether exposure to heatwave conditions can mitigate these acute effects, and the potential mechanisms underlying resilience, remain unknown. Resilience to heatwaves would require active molecular and physiological changes, such as alterations in gene expression^8,13^, which could in part, be influenced by epigenetic mechanisms such as DNA methylation^13–17^. However, it is unknown whether changes in DNA methylation can occur quickly enough to confer plasticity during rapid and intense climate events such as heatwaves.

Understanding the extent of tolerance to the interacting impacts of high temperature and hypoxia is particularly important for species of conservation concern, such as sturgeon, ancient fishes that are considered to be one of the most endangered group of vertebrates on the planet^18,19^. Many species of sturgeon can live over 100 years and thus these fish must be able to cope with changes in environmental conditions throughout their lives^20^. Thus, the ability to withstand episodes of thermal and hypoxic stress, or to induce plasticity to cope with these events, may play a key role in the survival of these iconic fish in a changing world.

In this study, we investigated the interacting effects of temperature and hypoxia on an endemic, endangered population of white sturgeon (*Acipenser transmontanus*)^21^ from the Nechako river in British Columbia (BC), Canada. This population is estimated to have declined from approximately 5,000 individuals to less than 600 spawning adults since the 1960s. Almost all wild sturgeon in the population are over 50 years old^22^, and there has been no recruitment in this population since the late 1960’s. Although adult sturgeon breed and produce embryos in the Nechako there is little or no survival of these juvenile fish during the first year of life^23^. Hatchery supplementation with juvenile fish released at one to two years of age is currently used to avoid extirpation of this population. To investigate the extent of resilience in response to heatwave conditions across levels of biological organization during their critical first year of life we used individuals from this supplementation program exposed to a simulated heatwave in the laboratory. Specifically, we asked:

1. Do juvenile sturgeon exposed to heatwave conditions exhibit cross tolerance to acute exposure to high temperature and hypoxia?
2. Do mRNA levels and global DNA methylation change over time during a heatwave?
3. Does exposing fish to heatwave conditions affect the plasticity of mRNA levels in response to an acute stressor?
4. Does acute exposure to high temperature and hypoxia induce rapid changes in global DNA methylation, and is this response different in fish exposed to a heatwave?

## Methods

### White sturgeon husbandry

In May 2021 gametes from wild-caught white sturgeon to be used as wild broodstock in the supplementation program were collected at the Nechako White Sturgeon Recovery Initiative (NWSRI) in Vanderhoof, British Columbia (54.0140° N, 124.0130° W). Eggs from two females were individually fertilized with sperm from each of three males in freshwater at 13 °C within 30 min of collection. It is important to note that this endangered population has limited adult individuals that are able to be used for hatchery spawning; 3 half-sib families were created from each female for six families in total. Immediately following fertilization embryos were randomly spread across six egg mats and within three minutes, the embryos were firmly adhered to the mats^24^. Embryos on egg mats were transported to the InSEAS aquatic facility at the University of British Columbia (UBC) in coolers, supplied with air stones to maintain oxygen levels and HOBO temperature loggers (Onset Computer Corporation; Bourne, MA, USA) to monitor temperature. Cool, dechlorinated water from the NWRSI was periodically added to coolers to maintain temperatures below 14 °C for the duration of the 10 h trip.

At UBC, embryos on egg mats were transferred to six 9L plastic tanks in a freshwater recirculating system (13 °C), and remained at this temperature until the start of the simulated heatwave. Once the sturgeon hatched and started feeding, freshly hatched artemia (Artemia International LLC; Texas, USA) were supplied three times daily. Juvenile sturgeon were also reared in the same six 9L tanks throughout this experiment. All fish in this study were reared and sampled under guidelines established by the Canadian Council for Animal Care and approved by the Animal Care Committee at the University of British Columbia under Protocol A19-0284.

### Heatwave exposure

**Figure 1:**
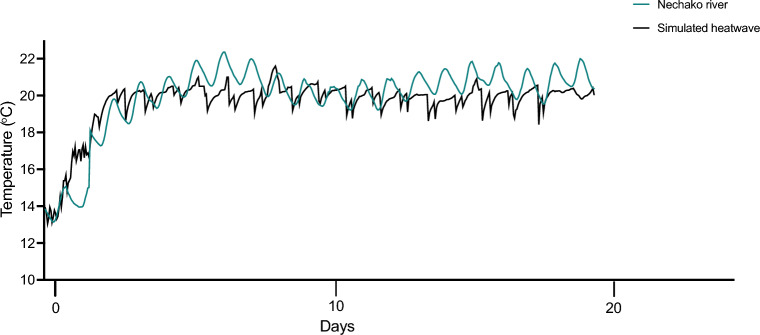
Nechako river temperature measured at the Vanderhoof station (teal) and recorded temperatures from the simulated heatwave (black). Temperatures taken during the British Columbia heatwave in June and July 2021.

In late June of 2021 an extreme heatwave occurred across British Columbia, including in the Nechako river, which is where the parental sturgeon were captured. We took advantage of the availability of near real-time temperature data from the Nechako river to perform an experiment to assess the resilience of white sturgeon to these heatwave conditions^25^. We exposed sturgeon to a simulated lab-based heatwave (Fig. 1) which allowed us to assess heatwave tolerance and subsequent resilience to other acute stressors during the life stage that would have been exposed to the heatwave in the wild.

**Figure 2:**
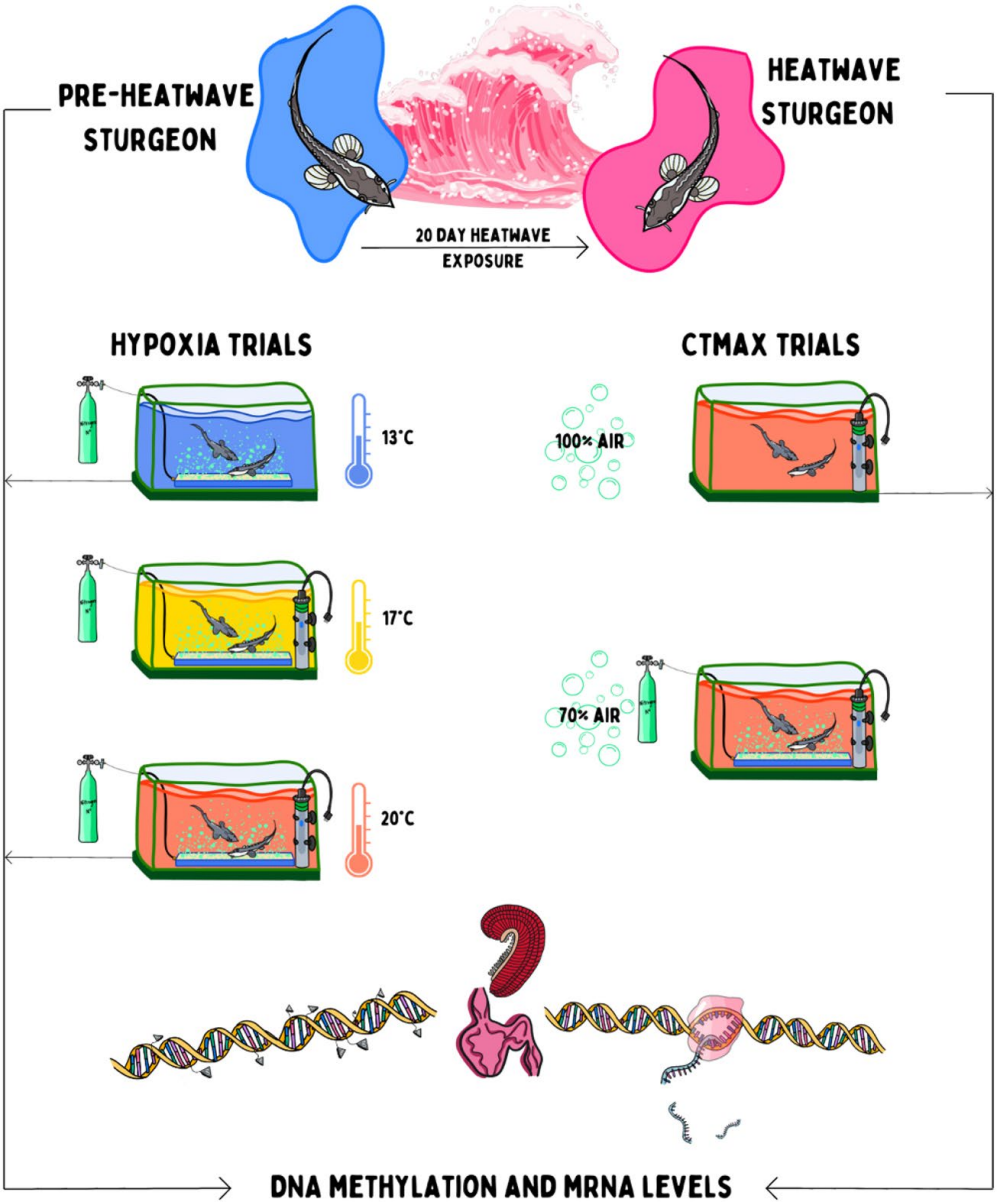
Experimental design for juvenile white sturgeon heatwave exposure. Juvenile sturgeon acclimated to 13 °C were exposed to a simulated heatwave mimicking the heatwave recorded in the Nechako river in June 2021. The laboratory exposures were performed in June through July of 2021 lagging a few days behind recorded river temperatures. This ensured that fish were exposed to the heatwave at the same life stage that they would have encountered these temperatures in the river. Temperature was increased to 20 °C over the course of three days and then maintained at that level for 20 days. Hypoxia and thermal tolerance (CTMax) were assessed in pre-heatwave (13 °C) fish and those exposed to 20 °C for 20 days. Hypoxia trials were conducted at the acclimation temperature and after acute exposure to 13 °C, 17 °C or 20 °C, acute increases to 17 °C and 20 °C for pre-heatwave fish and acute decreases 17 °C and 13 °C for heatwave fish. CTMax trials were conducted at either 70% or 100% air saturation. Sturgeon gills and heart were sampled at pre-heatwave, mid-heatwave (10 days), and heatwave (20 days) to assess baseline mRNA levels and global DNA methylation. Following hypoxia and CTMax trials stress induced levels of mRNA and global DNA methylation were also assessed (pre-heatwave and heatwave fish). Illustration by Madison Earhart.

To begin the heatwave exposure, temperature was increased with titanium aquarium heater sticks at a rate of 2.3 °C day^−1^ for three days, to simulate the rate of increase seen in the Nechako river during the 2021 heatwave (Fig. 1). Following these three days, water was at 20 ± 1 °C and juvenile white sturgeon were held at this temperature for 17 days, creating a 20-day heatwave in total. Temperature was recorded every 30 min with HOBO temperature loggers (Onset Computer Corporation; Bourne, MA, USA) throughout the experiment. Pre-heatwave (immediately prior to the onset of heatwave conditions) and after the heatwave (following heatwave exposure), thermal and hypoxia tolerance were assessed on a subsample of fish, and pre-heatwave, mid-heatwave, and at the end of the heatwave fish were sampled for assessment of mRNA levels and DNA methylation. We also assessed mRNA levels and DNA methylation following the acute thermal and hypoxia stressors in both pre-heatwave and heatwave exposed sturgeon (Fig. 2).

Due to the limited number of fish we were permitted to use from this critically endangered population, we did not have sufficient fish to hold a group of sturgeon at 13 °C throughout the trial. While this is a limitation of the experimental design, our goal was to assess the resilience of sturgeon to heatwave exposure and subsequent secondary stressors. Thus, comparisons between fish at the start of the experiment (at 13 °C) and at various times during the 20-day heatwave exposure should be regarded with caution as we cannot formally disentangle potential effects of 20 days of holding from the effects of heatwave exposure. However, prior work in our laboratory suggests that the measured traits are relatively stable to laboratory conditions during this life-stage with only 10 days separating our measurements.

### Thermal tolerance (CTMax) trials

Prior to and after the heatwave (Fig. 2) two CTMax trials were conducted, either under normoxic conditions, at 100% oxygen saturation (10.54 to 7.43 mg L^−1^), or under moderately hypoxic conditions, at 70% oxygen saturation (7.38 to 4.9 mg L^−1^). For every CTMax trial, 25 fish were randomly selected from the six tanks and placed in a mesh fish net breeder tank (Hagen, Montreal, Quebec, CA) in a 20 L experimental bucket containing aerated, circulating, dechlorinated water at the acclimation temperature of the treatment being tested (i.e. 13 °C or 20 °C). Fish were held in the experimental bucket for an hour prior to the trial to allow recovery from handling stress^26^. CTMax trials were conducted at the same time every day, between 10 am and 12 pm, to minimize diurnal effects on physiology^27^.

CTMax was assessed by increasing temperature by 0.3 °C min^−1^ until the fish lost equilibrium (LOE) and were unable to right themselves^26,28^. At the time of LOE, temperature was recorded and the fish was euthanized by immersion in an overdose of tricaine methanesulfonate solution (250 mg L^−1^; MS-222, Syndel Laboratory, Vancouver, Canada) buffered with an equal volume of sodium bicarbonate. Whole-body samples were then preserved in either 96% ethanol (DNA samples) or snap-frozen (RNA samples). For the normoxic CTMax trial (n = 25), oxygen levels were maintained at 95 ± 2% air saturation or higher by bubbling air through a porcelain gas diffuser, continuously monitored using a ProDO oxygen meter (Yellow Spring instruments, Yellow Springs, Ohio, USA). For the hypoxic CTMax trial (n = 25), nitrogen gas was introduced through a porcelain gas diffuser, acutely dropping oxygen at the rate of 1.5% min^−1^ until 70 ± 2% air saturation was reached. As the absolute concentration of oxygen dissolved in water is different between temperatures of 13 °C and 20 °C, the oxygen concentration for pre-heatwave trials started at 7.38 mg L^−1^ and ended at 5.48 mg L^−1^ and heatwave trials started at 6.37 mg L^−1^ and ended at 4.9 mg L^−1^. The nitrogen flow rate was adjusted throughout the trial to compensate for temperature effects on oxygen saturation.

### Hypoxia tolerance trials

Hypoxia tolerance was assessed as the oxygen level at LOE following acute exposure to declining oxygen levels. All trials were conducted at the same time each day between 10 am and 12 pm^27^. Fish were removed from their holding tanks and placed into a mesh breeder net (Hagen, Montreal, Quebec, CA) inside the experimental tank which contained 100L of aerated, dechlorinated, circulating water set to the respective acclimation temperature. Fish were permitted one hour to recover from handling stress prior to the trial. Oxygen was then lowered by bubbling in nitrogen to achieve a decline in dissolved oxygen of 1.5% min^−1^^29^. Both oxygen levels and temperature were continuously measured throughout the trial and nitrogen bubbling was manually adjusted to achieve the desired decline in oxygen. To test the effects of both acclimation and acute temperature exposure, independent hypoxia tolerance trials were conducted at 13 °C, 17 °C and 20 °C. Thus, pre-heatwave fish were tested at their acclimation temperature (13 °C) or during acute exposure to increased temperatures of 17 °C or 20 °C. Heatwave fish were tested at their acclimation temperature (20 °C) or during acute exposure to decreased temperatures of 17 °C or 13 °C (Fig. 2).

For assessment of pre-heatwave fish, for each hypoxia trial, 50 fish were randomly selected from the six holding tanks (8–9 fish per tank) and added to a mesh breeder net with the water temperature at 13 °C. Depending on the trial, during the 1 h rest period prior to hypoxia tolerance assessment, temperature was either maintained at 13 °C, or acutely increased to either 17 °C or 20 °C at a rate of 0.3 °C.min^−1^ and maintained for the remainder of the trial with aquarium heater sticks. Once each fish reached LOE, they were removed from the tank, euthanized, and the whole-body was preserved in either 96% ethanol or snap-frozen, as previously described (n = 25 for each sampling method).

For assessment of heatwave fish, for each temperature trial, 30 sturgeon were randomly selected from the experimental tanks and hypoxia tolerance was assessed as described above. For those trials involving acute temperature exposure, water temperature was acutely decreased to either 17 °C or 13 °C using a recirculating chiller and ice packs at a rate of 0.3 °C min^−1^ during the hour prior to the trial and then maintained at that temperature for the rest of the trial. The hypoxia tolerance assay was then conducted and at LOE fish were sampled as described above (n = 15 for each sampling method).

### mRNA transcript abundance

Total RNA was extracted from the gills and heart using an RNeasy kit (Qiagen; Hilden, Germany). Total RNA concentration and purity was determined for each sample using a Nanodrop 2000c (Thermo Scientific). RNA samples were then stored at  − 80 °C until further use.

cDNA was synthesized from 1 μg of DNAse treated RNA with a qscript cDNA synthesis kit (Quantbio; Beverly, Massachusetts), according to the manufacturer’s instructions. Following synthesis, cDNA was diluted tenfold with nuclease-free water for subsequent real-time quantitative polymerase chain reaction (RT-qPCR) analysis. cDNA was then stored at  − 30 °C until further use.

RT-qPCR was performed in a total volume of 10 μl per well with Bio-Rad Sso Advanced Universal SYBR-green supermix (5 μl per sample), nuclease free water (3 μl per sample), primer (1 μl per sample; 0.1 μl primer in 0.9 μl nuclease free water), and 1 μl cDNA. We assayed four target genes, *hsp90a, hsp90b, hsp70 and hif1a,* which were selected for their known roles in acute thermal and hypoxia responses in fishes. Thus, we predicted that these genes would increase in expression following both heatwave acclimation and acute thermal and hypoxia stress^26,60^. We also assessed the expression of three potential reference genes in both tissues (*rpl7, rps8, and rps5*). Primers were designed using a white sturgeon transcriptome^30^ and all were assessed for secondary structures and non-target binding. Primer efficiency (Supplemental 1) was determined using a standard curve generated from serial 1:10 dilutions of pooled cDNA. Both no template and no reverse transcriptase controls were run on each qPCR plate, and no genomic DNA or other contamination was detected. All reactions were completed in an RT-qPCR machine (Bio-Rad CFX96) in a 96-well plate as follows: 2 min at 95 °C, 40 cycles of 15 s at 95 °C, 30 s at 60 °C, and 30 s at 73 °C, a decrease for 1 min to 60 °C and 30 s at 72 °C. Melt curves were determined by denaturation for 15 s at 95 °C, a decrease for 1 min down to 60 °C and then a gradual increase of 0.075 °C s^−1^ to 95 °C. Only reference genes that had stable expression across treatments were used in subsequent analysis (*rpl7* and *rps8* for gill and all three for heart). Amplification data were normalized to the geometric mean of expression of the stable reference genes and then analyzed using the 2^△△CT^ method; the control group used to normalize data was the pre-heatwave baseline expression group^31^.

### Global DNA methylation

Global DNA methylation was measured in baseline samples from gills and hearts in pre-heatwave fish, fish exposed to 1 week of the heatwave (mid-heatwave), and fish exposed to the entirety of the heatwave (heatwave). Additionally, global DNA methylation was assessed in samples following the CTMax and hypoxia trials in pre-heatwave and heatwave sturgeon. Gills and heart were dissected from fish preserved in ethanol under a dissecting microscope and DNA was then extracted using a DNAeasy blood and tissue extraction kit (Qiagen, Germantown, Maryland, USA). Total DNA purity and concentration was assessed by gel electrophoresis and using a Qubit fluorometer (Invitrogen). Global methylation was assessed using a 5-mC DNA ELISA Kit (Zymo Research, Irvine, California, USA). Samples were run in duplicate and a standard curve was generated on each plate; intra-assay variation was 4.33% and inter-assay variation was 7.03%. Following color development, absorbance was measured at 450 nm using a 96-well plate spectrophotometer (SpectraMax 190, Molecular Devices). Global DNA methylation levels were normalized using the sterlet sturgeon genome, as this is the only sturgeon genome available^32^.

### Statistical analysis

All statistical analyses were completed using GraphPad Prism 9 with a significance level of 0.05. Normality and homogeneity of variance were assessed using Shapiro–Wilks, Levene’s tests, and visual inspection of Q–Q plots, if assumptions were violated data were transformed. Following all ANOVAs, multiple comparisons tests were performed with a Tukey’s HSD test.

CTMax data were analyzed using Mann–Whitney rank tests to determine significance within a treatment (100% vs. 70%) as the data could not be transformed to meet assumptions for parametric testing.

**Figure 3:**
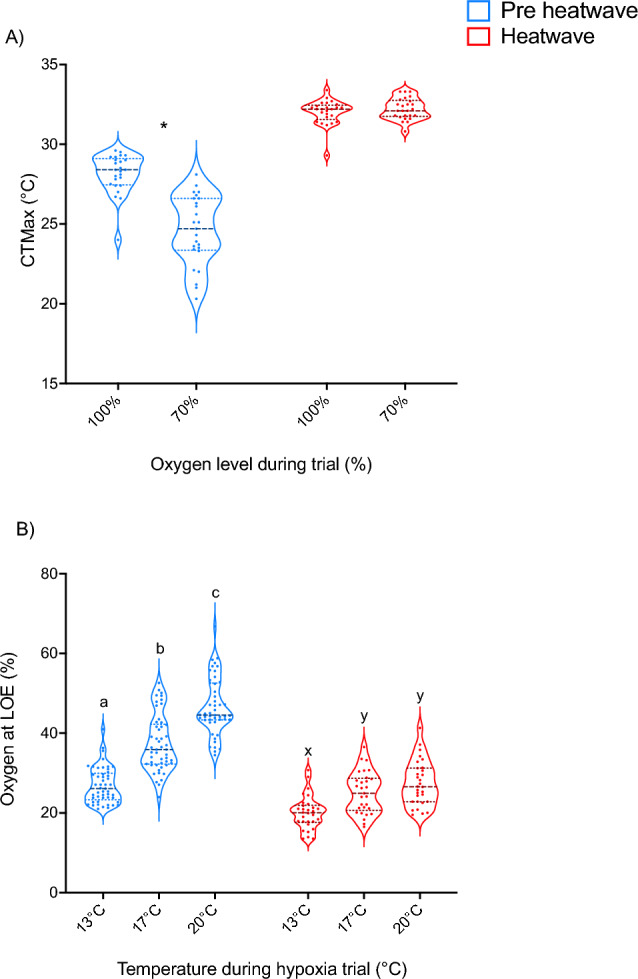
CTMax and hypoxia tolerance in pre-heatwave (pre-heatwave; blue) and heatwave exposed (heatwave; pink) juvenile white sturgeon. Panel (**A**) represents CTMax under normoxia (100% air saturation) or hypoxia (70% air saturation). Asterisks indicate CTMax differences between normoxic and hypoxic test conditions within pre-heatwave or heatwave fish respectively. Panel (**B**) shows % air saturation at the time of loss of equilibrium in pre heatwave and heatwave sturgeon at three different temperatures. Letters indicate differences in LOE between temperatures during the hypoxia trial, within each acclimation group. For both panels, data are expressed as a median with quartiles and individual data points are shown (n = 25–50).

Hypoxia tolerance data were log-transformed prior to analysis via two-way ANOVA with acclimation temperature (pre-heatwave or heatwave) and temperature during trial as fixed effects.

mRNA transcript levels for pre-heatwave, mid-heatwave, and heatwave timepoints were analyzed via one-way ANOVA. Baseline gill and heart global DNA methylation was also analyzed via one-way ANOVA.

**Figure 4:**
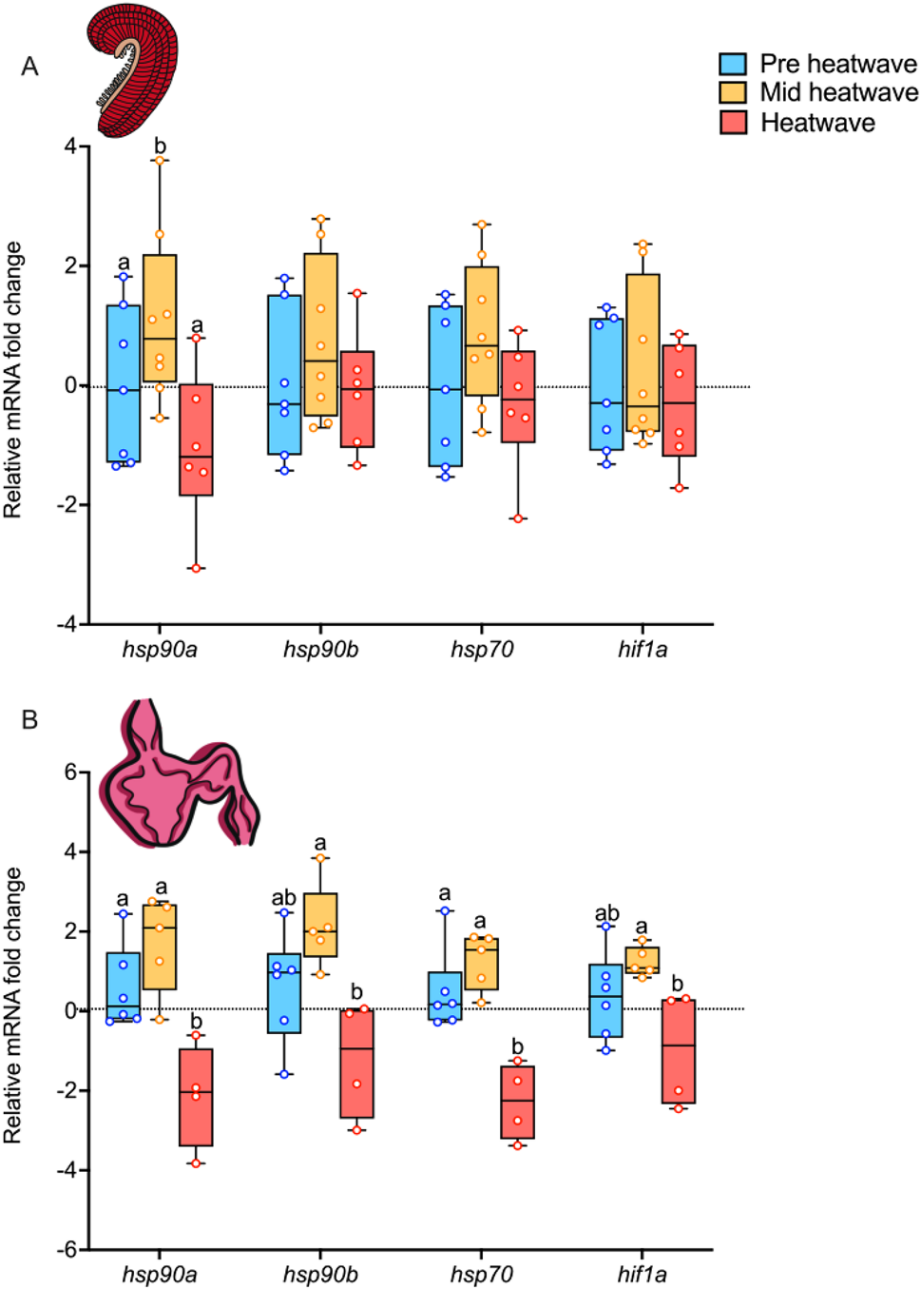
Baseline mRNA levels for *hsp90a, hsp90b, hsp70, and hif1a* in juvenile white sturgeon over time during the heatwave in fish that have not been exposed to an acute stressor. Data are normalized to the reference genes (see text) and presented as fold change compared to the mean of the control group (pre-heatwave). Pre-heatwave (blue), mid-heatwave (yellow), and heatwave (pink). For both panels, data are expressed as a median with quartiles and individual data points are shown (n = 5–8)*.* Panel (**A**) is gill mRNA and panel (**B**) is heart mRNA. Letters that differ indicate significant differences between pre-heatwave, mid-heatwave, or heatwave fish within a gene (*P* < 0.05).

**Figure 5:**
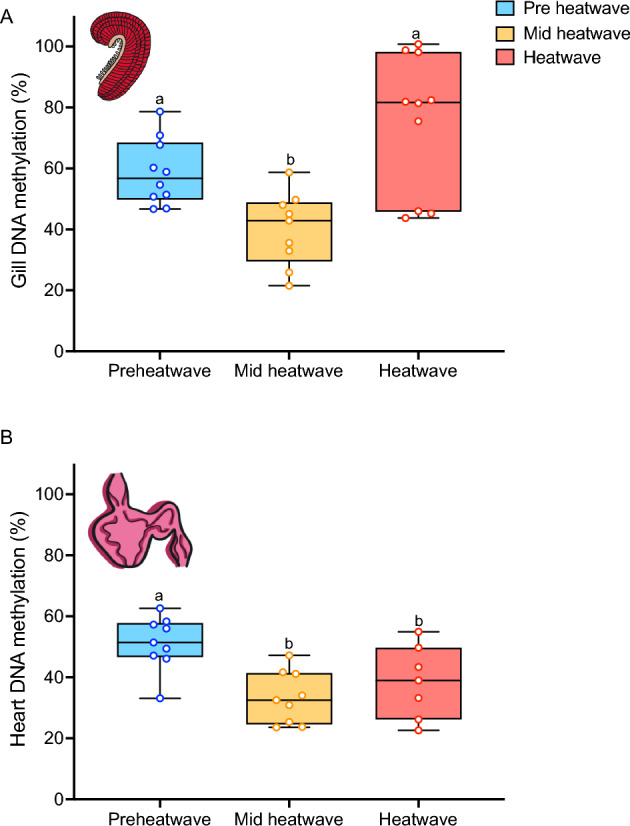
Baseline global DNA methylation levels in juvenile white sturgeon over time during the heatwave in fish that have not been exposed to an acute stressor. Pre-heatwave (blue), mid-heatwave (yellow), and heatwave (pink). Panel (**A**) is gill and panel (**B**) is heart. Letters that differ indicate significant differences between pre-heatwave, mid-heatwave, and heatwave fish as determined by Tukey HSD (*P* < 0.05). For both panels, data are expressed as a median with quartiles and individual data points are shown (n = 7–10).

Each gene’s mRNA level following CTMax and hypoxia exposure was analyzed via two-way ANOVA (with heatwave exposure and CTMax or hypoxia as factors). Both the gill and heart DNA methylation following CTMax and hypoxia were analyzed via two-way ANOVA (with heatwave exposure and CTMax or hypoxia as factors).

**Figure 6:**
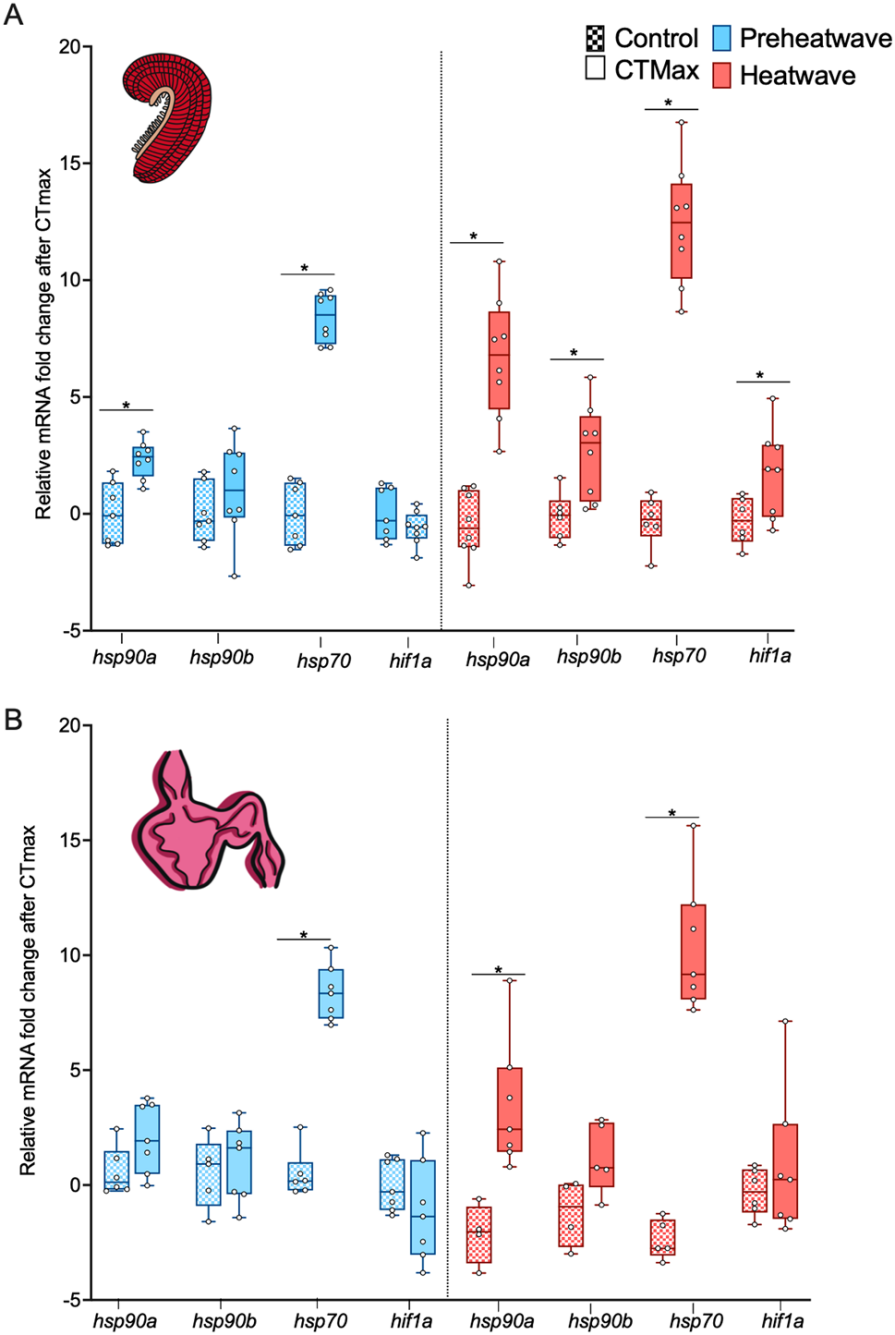
Fold change in mRNA transcript levels in unstressed fish (checkered boxes) and after CTMax trials (solid boxes) in pre-heatwave (blue) and heatwave (pink) juvenile white sturgeon. All mRNA levels are normalized to reference genes and fold change is computed relative to mRNA levels in unstressed fish for each gene. Panel (**A**) shows fold changes in gill mRNA and panel (**B**) shows fold changes in heart mRNA. An asterisk indicates differences in the fold change between unstressed fish and post-CTmax fish for each gene. Significance was determined via two-way ANOVA and a Tukey’s HSD (*P* < 0.05). For both panels, data are expressed as a median with quartiles and individual data points are shown (n = 4–8).

**Figure 7:**
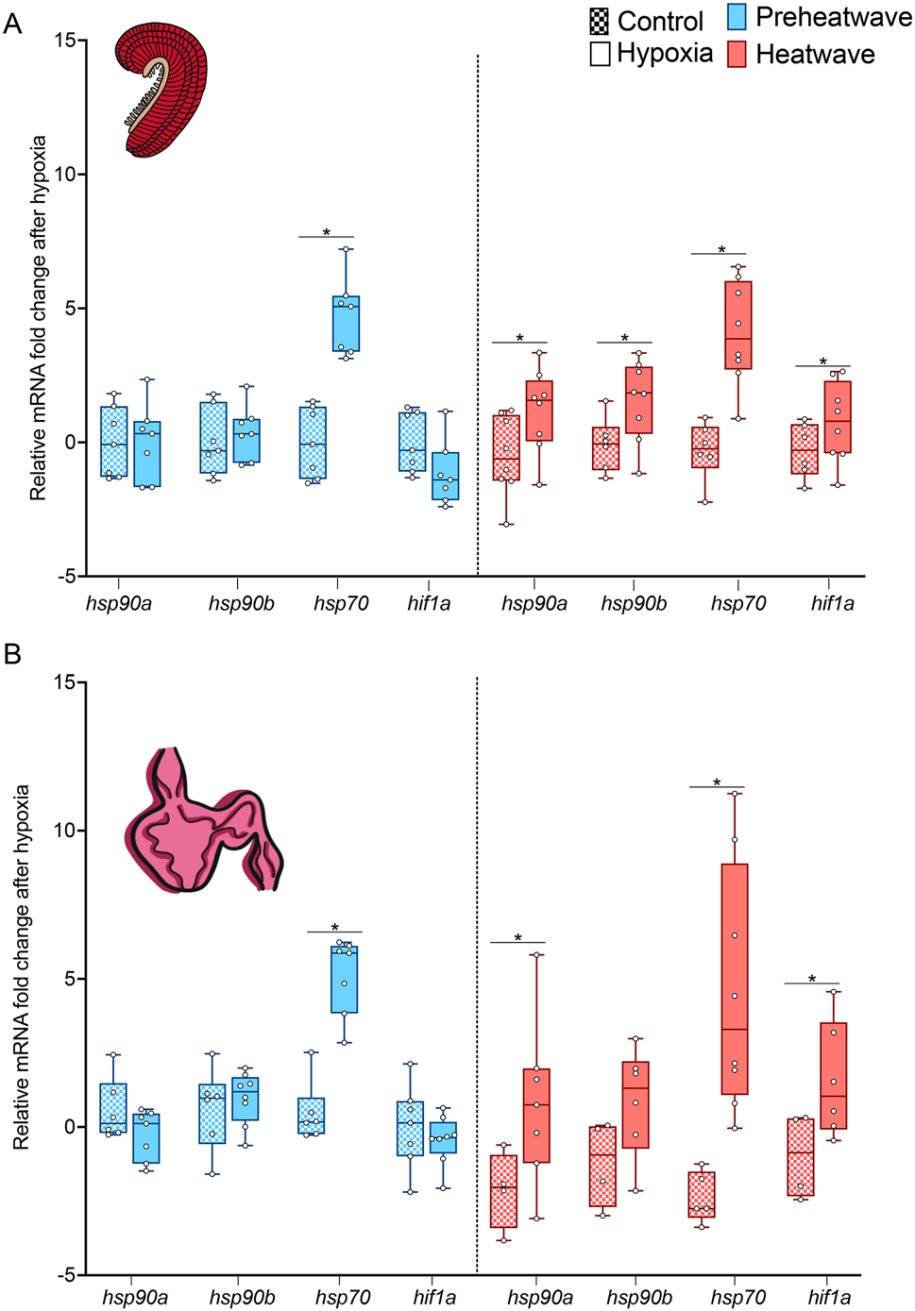
Fold change in mRNA transcript levels in unstressed fish (checkered boxes) and after hypoxia trials (solid boxes) in pre-heatwave (blue) and heatwave (pink) juvenile white sturgeon. All mRNA levels are normalized to reference genes and fold change is computed relative to mRNA levels in unstressed fish for each gene. Panel (**A**) shows fold changes in gill mRNA and panel (**B**) shows fold changes in heart mRNA. An asterisk indicates differences in the fold change between unstressed fish and post-hypoxia fish for each gene. Significance was determined via two-way ANOVA and a Tukey’s HSD (*P* < 0.05). For both panels, data are expressed as a median with quartiles and individual data points are shown (n = 4–8).

**Figure 8:**
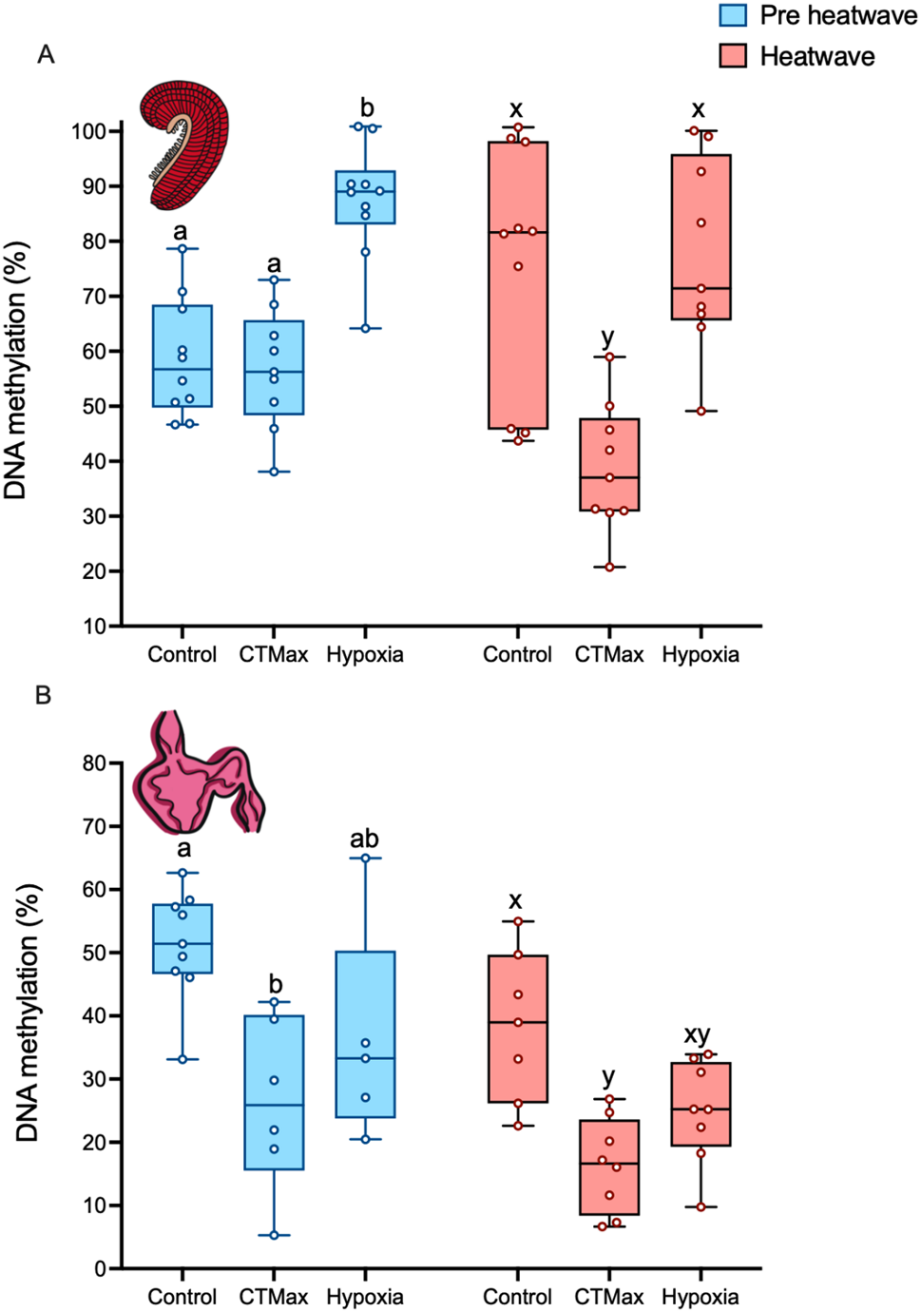
Gill and heart DNA methylation following CTMax and hypoxia trials in pre-heatwave (blue) and heatwave (pink) juvenile white sturgeon. Panel (**A**) shows gill DNA methylation and panel (**B**) shows heart DNA methylation. Letters that differ indicate differences between control, CTMax, or hypoxia DNA methylation within pre-heatwave (ABC) or heatwave fish (XYZ). Significance was determined via two-way ANOVA and a Tukey’s HSD (*P* < 0.05). For both panels, data are expressed as a median with quartiles and individual data points are shown (n = 5–10).

### ARRIVE guidelines

This study is reported in accordance with ARRIVE guidelines.

## Results

### Heatwave exposed sturgeon exhibit cross tolerance

Prior to exposure to heatwave conditions (pre-heatwave), acute hypoxic exposure reduced CTMax (Fig. 3a; *P* < 0.0001), with CTMax declining from 28.2 °C to 24.6 °C. Sturgeon exposed to heatwave conditions had a higher CTMax regardless of oxygen level (*P* < 0.0001). After heatwave exposure, there was no difference in CTMax (*P* > 0.05) between fish tested in normoxia (32.02 °C) and acute hypoxia (32.22 °C). Thus, induced cross-tolerance offset the acute effects of moderate hypoxia (70% O_2_ saturation) on CTMax in heatwave exposed fish.

Both exposure to acute temperature increase during the hypoxia trial (*P* < 0.0001) and heatwave exposure (*P* < 0.0001) significantly affected hypoxia tolerance, with a significant interaction between these two factors (*P* < 0.001). Prior to heatwave exposure (pre-heatwave fish), oxygen saturation at LOE increased from 26.9% to 37.4% to 46.6% with acute exposure to temperatures of 13 °C, 17 °C, and 20 °C, respectively (*P* < 0.0001 for all comparisons), indicating severely diminished hypoxia tolerance at higher temperatures (Fig. 3b). Heatwave exposure offset these effects such that fish acutely tested at 20 °C had similar hypoxia tolerance to pre-heatwave fish tested at 13 °C (LOE at 26.9% vs. 27.4%; *P* > 0.9999). Thus, juvenile sturgeon exposed to heatwave conditions demonstrate complete compensation for the effects of acute high temperature exposure on hypoxia tolerance. In addition, we observed less than 1% mortality during heatwave exposure, which highlights the substantial resilience of juvenile sturgeon to heatwave exposure and exposure to secondary acute stressors.

### Transcript abundance and DNA methylation over time

In the gill, gene mRNA levels stayed stable over time (Fig. 4a), with an increase in only one gene, *hsp90a,* at the mid-heatwave time point followed by a decrease in expression at the end of the heatwave (*P* < 0.05). Gill DNA methylation showed the opposite pattern of change across time (Fig. 5a; *P* < 0.0005), decreasing from pre-heatwave to mid-heatwave (*P* < 0.05) and then increasing from mid-heatwave to heatwave, returning to pre-heatwave levels (*P* < 0.0005).

Heart mRNA transcript abundance changed over time (Fig. 4b), with an increase in transcript abundance at the mid-heatwave time point and a decrease in transcript abundance by the end of the heatwave, with significant effects detected by one-way ANOVA for *hsp90a* (*P* < 0.005), *hsp90b* (*P* < 0.01), *hsp70* (*P* < 0.005), and *hif1a* (*P* < 0.05). Global DNA methylation of the heart also changed over time (Fig. 5b; *P* < 0.005), but less so than in the gill. Mid-heatwave, DNA methylation decreased in the heart (*P* < 0.005). However, unlike in the gill, heart DNA methylation did not return to baseline at the heatwave timepoint (*P* < 0.05).

### Heatwave exposed sturgeon exhibit increases in mRNA transcript plasticity following thermal and hypoxia stress

There were significant effects of having been exposed to a CTMax test on the mRNA abundance of *hsp90a*, *hsp90b*, and *hsp70* but not *hif1a* (Supplemental 2). Heatwave exposure significantly affected the abundance of *hsp90a, hsp70*, and *hif1a*, with a significant interaction between these factors for all genes except *hsp90b* (Supplemental 2). In general, fish exposed to heatwave conditions had larger increases in mRNA abundance in response to CTMax, such that heatwave fish had significantly higher mRNA abundance than their relative control levels as detected by post-hoc tests for *hsp90a*, *hsp90b, hsp70*, and *hif1a* (Fig. 6a). These data suggest that heatwave exposed sturgeon exhibit increased gill transcriptional plasticity in response to acute temperature stress.

Generally, the changes in heart mRNA abundance following CTMax were smaller than changes in the gill (Fig. 6b). Two-way ANOVA detected significant effects of CTMax only on the mRNA abundance of *hsp90a* and *hsp70* and there was no significant effect of heatwave exposure on the mRNA abundance of any gene. However, there were significant interactions for the two factors for both *hsp90a* and *hsp70* (Supplemental 2), with heatwave exposed fish showing greater transcriptional response when compared to their relative controls.

Two-way ANOVA detected significant effects of hypoxia exposure on the mRNA abundance of *hsp90b* and *hsp70* in the gill*,* but no significant effects of heatwave exposure or interaction between these factors for any of the heat shock protein genes (Supplemental 2). By contrast, for *hif1a* there were no significant main effects but there was a significant interaction between them. Only *hsp70* increased significantly in response to acute hypoxia exposure during hypoxia trials in pre-heatwave fish. (Fig. 7a) In contrast, in fish exposed to the heatwave all genes were significantly upregulated after the hypoxia trial.

There were few changes in heart mRNA levels in response to acute hypoxic exposure during hypoxia tolerance trials. Only *hsp90a* and *hsp70* were detected as significantly hypoxia responsive by two-way ANOVA. No significant effects of heatwave exposure were detected for any gene and only *hsp90a* exhibited a significant interaction between acute hypoxia exposure and the heatwave (Fig. 7b; Supplemental 2). In pre-heatwave fish, only *hsp70* was upregulated in the heart following acute hypoxia. In fish exposed to the heatwave, *hsp90a, hsp70* and *hif1a* were significantly higher than their relative controls following hypoxia trials.

### Thermal and hypoxia stress induce rapid changes in global DNA methylation

In this study and others^14–17,33–38^, DNA methylation demonstrates responsiveness to warm temperature acclimation, but whether rapid changes in methylation occur in response to acute high temperature and hypoxia exposures, and whether heatwave exposure affects these responses is unknown in fishes. For gill DNA methylation, two-way ANOVA detected significant effects of CTMax (Fig. 8a; *P* < 0.0005), no significant effect of acclimation condition (pre-heatwave vs heatwave; *P* > 0.05), and a significant interaction between the two factors (*P* < 0.005). In pre-heatwave fish, acute exposure to high temperature during a CTMax trial did not affect gill DNA methylation, whereas in heatwave fish, CTMax exposure resulted in a rapid and significant decrease in gill DNA methylation.

Similarly, there was a significant effect of hypoxia exposure (*P* < 0.01), no significant effect of heatwave exposure (*P* > 0.05), and a significant interaction between these factors (*P* < 0.05) on global gill DNA methylation. In pre-heatwave fish, gill DNA methylation increased from 56.43% to 73.32% (Fig. 8a) in response to acute hypoxia exposure, whereas it was high (75.35% to 77.74%) in heatwave fish both under normoxic conditions and after acute hypoxic exposure (Fig. 8a).

In the heart, DNA methylation was significantly affected by both CTMax (*P* < 0.0001) and heatwave exposure (*P* < 0.01), with no interaction between the factors. In both pre-heatwave and heatwave fish, CTMax exposure caused a statistically significant decrease in heart DNA methylation (Fig. 8b; pre-heatwave: 51.26% to 26.27% and heatwave: 38.42% to 16.32%). Similarly, heart DNA methylation was significantly affected by both acute hypoxia (*P* < 0.005) and heatwave exposure (*P* < 0.01) with no interaction between the factors. In general, heart DNA methylation was lower following acute hypoxic exposure in both pre-heatwave and heatwave conditions although this effect was not detected in post-hoc tests (Fig. 8b; pre-heatwave: 51.26 to 36.3% and heatwave: 38.42% to 24.9%).

Together, these data demonstrate the ability of DNA methylation to rapidly change in response to both acute temperature increase and hypoxia exposure, highlighting tissue-specific modifications that are enhanced by heatwave exposure.

## Discussion

In this study we demonstrate that juvenile white sturgeon exposed to heatwaves are resilient to this stressor during this critical life period. The heatwave exposed sturgeon exhibited cross-tolerance to subsequent acute stressors associated with changes in epigenetic marks and mRNA levels in response to acute thermal and hypoxia stress. Of particular note, sturgeon demonstrate the capacity to rapidly change global DNA methylation in response to acute heat or hypoxic stress over the course of a few hours. This study illustrates the extent of, and mechanisms underlying, resilience in juvenile white sturgeon in response to climate change scenarios during their most vulnerable year of life. Ultimately, these physiological mechanisms may have significant benefits in dealing with the increases in heatwave duration and frequency associated with climate change.

### Exposure to heatwave conditions induced cross-tolerance to high temperature and hypoxia

As expected, based on many studies of the effects of thermal acclimation on thermal tolerance in fishes^39^, exposure to increased temperature during the simulated heatwave resulted in an increase in thermal tolerance in juvenile sturgeon. Although the observed differences in CTmax between pre-heatwave and heatwave fish could, in principle, be due to the 20 days between these two measurements, it is much more likely that this increase in thermal tolerance is due to thermal acclimation associated with the heatwave exposure. Not only is it well documented across fish species that warm acclimation increases thermal tolerance^8,39^, this response to thermal acclimation has also been reported in the same population of sturgeon at different ages^28,40,41^. Increases in CTmax following various thermal acclimation temperatures are demonstrated across white sturgeon populations^42, 43^ and other sturgeon species^26,28,40,43–46^. In fact, sturgeon have higher capacity for acclimation, measured as the acclimation response ratio (ARR), and show unusual plasticity and resilience to rapidly increasing temperatures compared to other fishes^39,47^. Further, this acclimation capacity is even more pronounced in early developmental stages, where white sturgeon at the yolk-sac larvae stage have an ARR of over 1^40^. In this study, juvenile sturgeon exhibited an ARR of 0.55, which is higher than average for a temperate fish species^39^, but similar to that of slightly older white sturgeon^28^ indicating that white sturgeon have substantial capacity for plasticity and are remarkably tolerant to temperature changes.

Much less studied is the possibility that thermal acclimation also results in cross tolerance to hypoxia^8,12^, a phenomenon that occurs when the mechanisms that are enhanced to protect against one stressor also elicit protection against a second stressor^8^. Here, we demonstrate clear evidence of cross-tolerance in juvenile sturgeon after heatwave exposure highlighting the resilience of this species in response to extremely stressful environmental conditions. Importantly, sturgeon were able to completely compensate for the negative effects of acute temperature increases on hypoxia tolerance. Prior to the heatwave, acute exposure to 20 °C resulted in a near-doubling of oxygen saturation at LOE compared to fish tested at 13 °C (i.e., acute high temperature exposure greatly reduced hypoxia tolerance). In fish exposed to heatwave conditions, hypoxia tolerance at 20 °C was not different from that of pre-heatwave fish tested at 13 °C, indicating complete compensation of this trait^48^. In comparison, most fish species demonstrate little or no compensation of hypoxia tolerance with warming, and if they do, compensation is generally only partial and only occurs across a relatively small range of temperatures^49,50^. Juvenile sturgeon thus exhibit a much greater degree of cross-tolerance than has been demonstrated for most fish species. The extent of cross-tolerance is also evident from the observation that heatwave exposed sturgeon had the same CTMax under both normoxia and hypoxic conditions. Thus, heatwave exposed fish were able to completely compensate for the negative effects of moderate hypoxia on thermal tolerance. Although we cannot formally rule out the possibility that the observed differences between heatwave exposed and pre-heatwave fish were due to the 20 days in the laboratory, given that thermal acclimation has been shown to result in cross-tolerance to hypoxia in at least some fish species^8,51^, we suggest that the observed cross-tolerance in heatwave exposed fish represents a plastic response. It is notable that these results are based on exposure to temperatures mimicking recorded river temperatures, whereas most previous work has been performed using constant laboratory exposures. These results indicate that sturgeon during their first year of life have the ability to accrue cross tolerance to withstand environmentally realistic multi-stressor challenges.

### Exposure to heatwave conditions causes long-lasting changes in mRNA levels and global DNA methylation

The underlying mechanisms that induce resilience and cross-tolerance following thermal acclimation are poorly understood in fishes^8,13^. In this study we measured baseline mRNA levels for several key genes predicted to be involved in the response to acute high temperature and hypoxic exposure before (pre-heatwave), during (mid-heatwave), and after (heatwave) exposure to heatwave conditions. In all genes measured in the gill, transcript abundance showed a similar pattern over time, with only *hsp90a* increasing halfway through the heatwave but returning to baseline levels following the entirety of the heatwave exposure.

In the heart, genes had an increase in mRNA levels halfway through the heatwave exposure period and a decrease in levels, back to pre-heatwave levels or below, by the end of the heatwave^52^. While this could be due to the 10 days between measurements, it is much more likely this change in baseline mRNAs is a result of heatwave exposure. Increases in heat shock protein (*hsp)* and hypoxia-inducible-factor-1 (*hif1a)* transcript abundance are a common response to thermal stress in fishes^52–55^, which suggests that mid-heatwave the sturgeon may have been thermally stressed. By the end of the heatwave mRNA levels returned to or below pre-heatwave levels, and this decrease in abundance over time could indicate chronic thermal stress or complete acclimation to a warmer temperature^13,52,56^. Paired with the observed changes in whole animal tolerances, which exhibit substantial compensation, and low levels of mortality (less than 1%) throughout the heatwave, we suggest that the changes in mRNA levels after 20 days of chronic heatwave exposure reflect a completed acclimation response.

We also measured global DNA methylation at the same time points to assess whether this regulatory mechanism influencing mRNA abundance also changed over the course of the 20-day heatwave exposure. Changes in DNA methylation during warming have been observed across many organisms from polychaetes^33^, to coral^38,57^, to teleost fishes^16,35–37,39^ and here we show this pattern also occurred in white sturgeon, an ancient fish species. Thus, we suggest that while it may be possible these differences are due to the 10 days in between measurements, it is much more likely these changes are due to heatwave exposure. However, it should be noted that the whole-genome methylation levels in juvenile sturgeon are lower than what is typically reported in teleost fishes^58^, although still much higher than is typical for invertebrates^59^, suggesting a fundamental difference in the role of DNA methylation as an epigenetic regulatory mechanism across taxa.

In this study, gill methylation levels decreased mid-heatwave. Generally in vertebrates, decreases in methylation are associated with increases in transcription activity, and thus this pattern is suggestive of an overall increase in transcription^16^ in response to increased temperatures. Methylation returned to baseline levels by the end of the heatwave, possibly signifying complete tolerance to the increased temperature. Conversely, heart DNA methylation decreased mid-heatwave and stayed at this level through the end of the heatwave, demonstrating tissue-specific responses of DNA methylation to heatwave exposure. We further suggest that the remarkable physiological resilience that white sturgeon exhibit in response to a heatwave is, at least in-part, influenced by changes in molecular underpinnings such as transcriptional activity and DNA methylation.

### Heatwave exposed sturgeon exhibit substantial plasticity of mRNA levels in response to an acute stressor

The observed changes in whole-organism physiology in response to acute thermal and hypoxic stressors (e.g., increases in CTMax and decreases in oxygen saturation at LOE) in response to heatwave exposure in juvenile sturgeon were associated with a plastic transcriptional response following exposure to acute warming. An increase in gill *hsp* transcriptional activity after acute thermal stress following warm acclimation has also been demonstrated by other animals^26,56,60^, including sturgeon, suggesting high temperature acclimation may increase future thermal tolerance through increases in gill transcriptional plasticity. In the heart, changes in mRNA transcript abundance were not as responsive to acute thermal stress as in the gill; but the heatwave exposed sturgeon had increased plasticity in the mRNA levels of both *hsp90a* and *hsp70*. Similarly, in other organisms both the heart and gill increase *hsp* transcription after heat stress^61–63^, but with varying levels of plasticity in the gill versus the heart depending on the species and duration and intensity of heat stress. The findings presented here and across other fish species suggest that increasing transcriptional plasticity in response to acute temperature change following thermal acclimation is a common defense strategy. As such, climate events like heatwaves may increase juvenile sturgeon tolerance when they encounter future acute temperature changes.

Interestingly, there were differences in the extent of trait variation among individuals between pre-heatwave and heatwave exposed fish. For CTmax, pre-heatwave fish exhibited much more variation than did fish exposed to the heatwave. This pattern suggests that there could be inter-individual variation in the extent of plasticity in response to heatwave exposure. It is also consistent with the idea of “plastic floors and concrete ceilings”^64^ which suggests that upper thermal tolerance measured as CTmax has a relatively fixed upper limit above which no further plasticity can be induced. We hypothesize that heatwave exposed sturgeon are reaching this ceiling and as a result individuals with relatively low pre-heatwave CTmax express greater plasticity than individuals with relatively high starting CTmax. In contrast, we do not observe this pattern of reduced variation post-heatwave at the molecular level under baseline conditions. Indeed, variance in gene expression levels were greater in heatwave exposed fish following CTmax exposure. This suggests a lack of strict coupling across these levels of biological organization, although additional experiments would be necessary to rigorously test this idea. Together these differences at the molecular and whole-organism levels highlight the complexity of making functional linkages across biological levels of organization.

The ability of the juvenile sturgeon to fully compensate for the negative effects of high temperature on hypoxia tolerance following heatwave exposure is very rare in fishes^8,65^ and it is likely that transcriptional plasticity is playing a role in this phenomenon. Exposure to acute hypoxia resulted in substantial (~ fivefold) increases in *hsp70* levels in both gill and heart in pre-heatwave and heatwave sturgeon*.* Increases in *hsp70* abundance in response to hypoxia have also been demonstrated in other sturgeon species and thus induction of *hsp70* appears to be a common response to hypoxic stress in sturgeon^66,67^ and other fishes^68,69^.

Acute hypoxia stress resulted in substantial increases in *hif1a* in sturgeon that were exposed to the heatwave, which could be partially influencing the hypoxia tolerance demonstrated by these fish. *Hif1a* is a subunit of a transcription factor that regulates the expression of genes involved in metabolism and oxygen supply, and is known to orchestrate the cellular response to hypoxia^70,71^. In other aquatic animals, the mRNA abundance of *hif1a* changes in response to thermal acclimation^26,72^ and subsequent or co-current hypoxia exposure^73,74^, but the effect these changes have on cross-tolerance differs among species. Regardless, in this study, the heatwave exposed sturgeon demonstrated complete compensation of hypoxia tolerance and increased *hif1a* mRNA plasticity during the hypoxia trial, highlighting the contribution *hif1a* may have in promoting this cross-tolerance.

### Acute exposure to high temperature and hypoxia induces rapid changes in global DNA methylation, and this response is enhanced in heatwave exposed sturgeon

For the first time in fish, we demonstrate rapid changes in global DNA methylation over the course of an hour in response to acute thermal and hypoxia stress. Not only do we see rapid plasticity in methylation, these changes are tissue-specific, different between stressors, and different between pre-heatwave and heatwave exposed fish. In response to acute thermal stress, heatwave fish rapidly decreased gill DNA methylation to half that of baseline levels. By contrast, DNA methylation levels were not affected by acute high temperature exposure of pre-heatwave fish. The rapid decrease in global gill DNA methylation in heatwave-acclimated fish suggests that they may be able to recruit greater transcriptional responses to acute thermal stress, which is consistent with our observation of increased mRNA plasticity in gill following CTMax in heatwave exposed sturgeon. In the heart, global DNA methylation decreased in response to acute high temperature exposure in both heatwave-acclimated and pre-heatwave fish, highlighting the similarities and differences between these tissues.

Acute hypoxia exposure was associated with high levels of global DNA methylation in the gill. In the pre-heatwave fish, this was the result of an increase in DNA methylation as a result of acute hypoxic exposures, whereas in the heatwave exposed fish, DNA methylation was high under normoxic conditions, and remained so following acute hypoxia exposure. This pre-conditioning of global DNA methylation also has the potential to play a role in the observed cross-tolerance of the heatwave exposed fish.

Contrasting patterns of DNA methylation in response to acute hypoxic exposure were observed in the heart, where DNA methylation slightly decreased from baseline levels in response to acute hypoxia exposure in both heatwave and pre-heatwave fish. Similar patterns of decreases in heart DNA methylation in response to hypoxia have been observed in other fish species^75^, suggesting that this may represent a common response to hypoxic stress in fish hearts.

The difference in DNA methylation in response to thermal and hypoxia stress in the gill may indicate opposing cellular strategies to cope with these stressors. The gill is the interface between the external and internal environment and hypoxic conditions are known to damage this tissue^70^. As high levels of DNA methylation are often associated with decreases in transcriptional activity in vertebrates, this would be consistent with the observation of decreases in transcriptional activity in response to cellular stress^55^. Further, in this study and others hypermethylation has been demonstrated in response to environmental stressors such as thermal stress^16,76^. It has been suggested that an inactivation of generalized gene expression may conserve energy for activation of stress-responsive genes, and as such a hyper-methylated state may be beneficial for stressor tolerance. The heart, however, shows an opposite pattern, suggesting that there may be different thresholds for suppression of transcriptional activity to environmental stress across tissues.

## Conclusion

The collective evidence from this work demonstrates that juvenile white sturgeon exhibit substantial resilience in response to heatwave conditions during a critical life stage. Heatwave-exposed sturgeon exhibit a high level of cross-tolerance, in whole animal physiological responses to acute thermal and hypoxia stress. The complete compensation observed during acute thermal and hypoxia exposures is unusual among fish species and is clear evidence of the impressive tolerance of white sturgeon.

The observed heatwave resilience in juvenile white sturgeon was associated with changes in mRNA levels and DNA methylation. White sturgeon demonstrated molecular plasticity, including a rapid induction of changes in DNA methylation in response to acute stressors. This is the first study to demonstrate these rapid changes in methylation in fishes as a response to acute thermal or hypoxia stress. These data highlight a previously unappreciated mechanism of thermal compensation, but questions remain concerning whether this capacity is unique to sturgeon and associated with the fact that they are an ancient, long lived, polyploid species.

Lastly, these findings have environmentally relevant consequences for understanding the thermal sensitivity of a critically endangered species during their most vulnerable year of life. As heatwaves are one of the largest threats to global biodiversity, it is encouraging that, as demonstrated in this study, this iconic, critically endangered white sturgeon population has the physiological capacity to cope with extreme thermal stress. This study highlights the resilience of white sturgeon to heatwave events, and emphasizes the importance of measuring animal tolerance to extreme climate change events using realistic thermal exposures in endemic, endangered species.

### Supplementary Information

Supplementary Information 1.

Supplementary Information 2.

## Data Availability

Supplemental figures and tables for this article can be found in the electronic supplementary material. All data is available here doi: 10.5061/dryad.n5tb2rc0h.
