# Supplementary material for: Heatwave resilience of juvenile white sturgeon is associated with epigenetic and transcriptional alterations
Source: Sci Rep. 2023 Sep 18;13:15451. doi: 10.1038/s41598-023-42652-7 (PMC10507091; doi:10.1038/s41598-023-42652-7)
Supplement: Supplementary file 1 — Supplementary Information 1. [file 41598_2023_42652_MOESM1_ESM.docx]

Supplemental 1. Primer sequences for white sturgeon, *Acipenser transmontanus*, *HSP70, HSP90a, HSP90b, HIF1a, RPL7, RPS8, and RPS5.*

| Gene | Forward primer | Reverse primer | Efficiency (%) |
| --- | --- | --- | --- |
| *HSP70* | CCATGAACCCCAGCAACACT | TGCACAACAGAGTCGTCGTA | 100.7 |
| *HSP90a* | CCTTGATTGCCTCCTCTGTT | GACTCATTCCAACCGCATCTA | 103.4 |
| *HSP90b* | ACTTGGTCCTTGCTCTCACC | GCGATACCACAGCTCTCAGT | 104.9 |
| *HIF1-a* | GCATCTGAGGATAGTGGTAAAG | CTGTTGGCAGTAGGAGAATG | 99.5 |
| *RPL7* | TCACAAAGGCCAACTTGGGT | CGAGATCCGCTTGTCCAGAA | 101.9 |
| *RPS8* | GGGCGACCCAATTCATACTT | CCAGGGACAACTGGCATAAA | 102.8 |
| *RPS5* | ACTCGACCCGAATTGGACG | GTTGACTCTACGCAGGGGG | 99.9 |
